# Scalable graphene sensor array for real-time toxins monitoring in flowing water

**DOI:** 10.1038/s41467-023-39701-0

**Published:** 2023-07-13

**Authors:** Arnab Maity, Haihui Pu, Xiaoyu Sui, Jingbo Chang, Kai J. Bottum, Bing Jin, Guihua Zhou, Yale Wang, Ganhua Lu, Junhong Chen

**Affiliations:** 1 Department of Mechanical Engineering, College of Engineering & Applied Science, University of Wisconsin-Milwaukee, Milwaukee, WI 53211 USA; 2 Pritzker School of Molecular Engineering, University of Chicago, Chicago, IL 60637 USA; 3 Chemical Sciences and Engineering Division, Physical Sciences and Engineering Directorate, Argonne National Laboratory, 9700 S. Cass Ave., Lemont, IL 60439 USA

**Keywords:** Nanosensors, Two-dimensional materials

## Abstract

Risk management for drinking water often requires continuous monitoring of various toxins in flowing water. While they can be readily integrated with existing water infrastructure, two-dimensional (2D) electronic sensors often suffer from device-to-device variations due to the lack of an effective strategy for identifying faulty devices from preselected uniform devices based on electronic properties alone, resulting in sensor inaccuracy and thus slowing down their real-world applications. Here, we report the combination of wet transfer, impedance and noise measurements, and machine learning to facilitate the scalable nanofabrication of graphene-based field-effect transistor (GFET) sensor arrays and the efficient identification of faulty devices. Our sensors were able to perform real-time detection of heavy-metal ions (lead and mercury) and *E. coli* bacteria simultaneously in flowing tap water. This study offers a reliable quality control protocol to increase the potential of electronic sensors for monitoring pollutants in flowing water.

## Abstract (web summary)

2D biochemical sensors hold potential for monitoring toxic contaminants in water, but they are usually affected by device-to-device variations. Here, the authors propose a scalable fabrication process and quality control procedure to realize graphene field-effect transistor arrays able to simultaneously detect trace amounts of heavy metal ions and bacteria in flowing water.

## Introduction

Inadequate management of drinking water exposes hundreds of millions of people worldwide to dangerous contaminants^1,2^ which can threaten public health and lead to the transmission of various diseases such as diarrhea and cancer^3^. The United Nations’ Sustainable Development Goals call for universal and equitable access to safe and affordable drinking water that is free from fecal (e.g., bacteria) and priority chemical contaminations (e.g., heavy metals) by 2030^4^. Therefore, there is a growing need to identify potential health hazards in water to provide early warning and prevent catastrophic events, which requires intelligent, fast, adaptable, and continuous sensing systems to forecast water contamination. Compared to the time-consuming, expensive, and bulky experimental setups of state-of-the-art mass-spectrometry-based techniques which limit their capabilities for continuous online monitoring, electronic sensors show promise in accomplishing this task due to their superior performance (e.g., rapid response, high sensitivity and selectivity, low cost, and easy operation) and potential for integration with existing water infrastructure and wireless data transmission^5^.

Two-dimensional (2D) layered nanomaterial-based field-effect transistors (FETs) have been successfully demonstrated for chemical and biological sensing (e.g., heavy-metal ions, gas/bio-molecules, and bacteria)^6–19^. However, commercialization of 2D FET sensors^20^ for real-time water sensing still remains challenging for scale-up fabrication, primarily because of poor device quality control, leading to device-to-device variations in response trends, calibration, and reliability. Current attempts to address these issues have largely focused on the prerequisite step of controlling the sensor’s channel materials, including the large-scale chemical vapor deposition growth for 2D nanomaterials^21–23^, printing their thin films directly^24–27^, spin-coating, and self-assembly on the substrate^28–30^. Among these, the wet transfer of 2D layered nanomaterials in the solution phase onto the substrate by spin-coating can be an efficient, versatile, and rapid approach for the large-scale nanofabrication of electronic devices^30^. Nevertheless, identifying a single sheet of nanomaterial and the subsequent patterning of a single pair of electrodes in a FET sensor is a tedious and energy- and cost-intensive effort. In contrast, the parallel connection of multiple flakes by interdigitated electrodes can scale-up fabrication of 2D FETs more quickly while reducing power consumption and thus commercialization costs. However, there is not yet a holistic approach available that can compensate for device variations by directly correlating the faulty sensor devices with non-destructive measurements to isolate them in the large-scale manufacturing process, nor in modeling the sensor responses of ideal-like devices with advanced data analysis to attain highly accurate predictions.

Here, we report on the strategic control of the quality of 2D FET sensor devices during scale-up fabrication using a bottom-up approach that enables reliable and real-time monitoring of toxins in flowing water, as demonstrated in a graphene-based field-effect transistor (GFET) sensor array. Heavy metals (lead and mercury) and *E. coli* bacteria were selected as representative pollutants for testing because they are major contaminants in drinking water supplies. Technically, wafer-scale sensor devices were first fabricated by the wet transfer of single-layer graphene oxide (GO) dispersion in water and the subsequent patterning of interdigitated electrodes to pre-screen uniform devices by their electronic properties. We found that a majority (~60%) of devices after thermal annealing achieved a relatively narrow electronic distribution (variation within ±10% with respect to the mode value) for both the resistance and the drain current on/off ratio. This initial fabrication process with narrow distributions of electronic properties is only a prerequisite step for obtaining a uniform response tendency from sensor devices. Faulty devices were then successfully identified by correlating the non-ideal response behaviours (i.e., bidirectional) with the measured impedance ratio of Zˊ/Z˝ > 1000 at low frequencies, which is likely attributed to optically invisible defects in the top dielectric layer (3-nm Al_2_O_3_). The drain current noise power spectral density (PSD) further indicated the absence of any significant types of defect traps in the as-deposited Al_2_O_3_ in near-ideal sensor devices after pre-screening and validated the effect of chemical gating from the surface adsorption. The responses of the GFET sensor array for the simultaneous detection of selected heavy metals and bacterial species in flowing water were finally calibrated by machine learning (ML) modeling with high-precision classification and quantification at the ppb (cfu/mL) level.

## Results

### Scalable nano-fabrication of GFET sensor array

The sensing signal from a FET sensor is typically transduced from the surface potential (i.e., gating effect) into change in the channel conductance $G$ (or resistance $R$) upon the surface adsorption of target analytes. The sensor response for multiple n-type/p-type semiconductor nanoflakes connected in parallel between a pair of source-drain electrodes can be described by $G$ (over $R$ for the simplicity of mathematical representation here) as

1 $$\frac{\Delta G}{G}=q\Delta Q\frac{{\sum }_{i}^{N}{\mu }_{i}{w}_{i}{h}_{i}}{{\sum }_{i}^{N}{\sigma }_{i}{w}_{i}{h}_{i}},$$

where $q$, $\Delta Q$ are the elementary charge and change in the concentration of major charge carriers, $\mu$, $\sigma$, $w$, and $h$ are the major carrier mobility, conductivity, lateral width, and height of an individual flake, respectively, and $N$ is the total number of flakes. Here, $\Delta Q$ is treated as constant in a global gating and sensing environment (i.e., the analyte-binding-induced gating effect is shared). Equation (1) can be reduced to that of a single flake device if both the intrinsic and extrinsic properties (i.e., $\mu$, $\sigma$, $w$, and $h$) are the same for all individual flakes. However, these properties are generally different and thus lead to device-to-device variations (especially for non-crystalline channel materials, while the extrinsic properties can still vary for crystalline materials). An effective way to reduce such variation is to increase the total number *N* of single flakes and combine them into an ensemble.

**Fig. 1:**
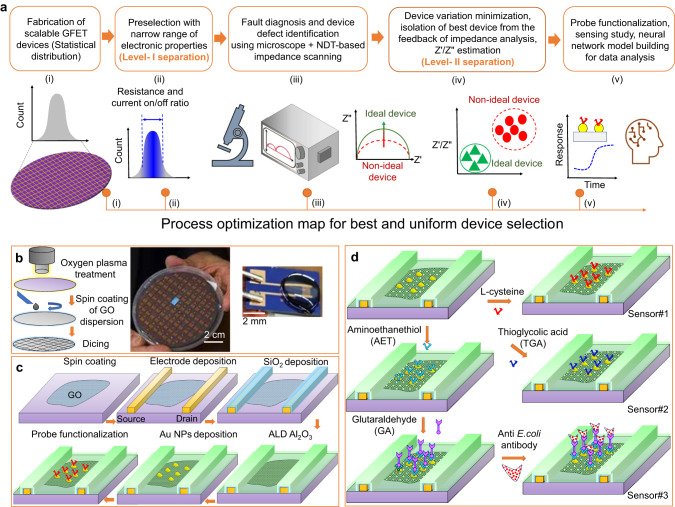
Wafer-scale nanofabrication and functionalization of the graphene-based field-effect transistor (GFET) sensor array. **a** Schematic nanofabrication strategy with device preselection (fault diagnosis) using impedance measurements to minimize device variation in order to achieve a near-ideal response pattern. Each step of the testing/analysis procedures is schematically shown (i-v) with the corresponding descriptions in the inset. **b** Spin-coating graphene oxide (GO) dispersion onto an entire wafer substrate for the deposition of patterned electrodes. The photographs show the wafer-size sensor devices after dicing and a single device with a droplet of test solution on the surface. **c** The nanofabrication steps of the rGO-based FET sensor device include electrode deposition by e-beam lithography, deposition of the SiO_2_ protection layer on the electrode using e-beam lithography, 3-nm atomic layer deposition (ALD)-derived Al_2_O_3_ acting as the top gate oxide, sputtering Au nano-particles (NPs) as anchoring sites for probes, and probe functionalization. **d** Schematic steps of the probe functionalization for Sensors 1-3 with specific biochemical and antibody probes.

Our strategy to advance the transition of FET sensors from proof-of-concept into real-world applications starts with large-scale device fabrication with quantified measures to mitigate device-to-device variations (Fig. 1a). To this end, wafer-size nanofabrication was carried out by spin-coating the channel materials in the solution phase to a wafer substrate and patterning interdigitated electrodes (100 pairs) for parallel connection, where monolayer GO dispersion in water was piloted as the precursor of the sensor channel (Fig. 1b). To eliminate the adverse effect of ion accumulation on the electrodes (due to the direct contact between the source-drain electrodes and water) and to enable the gating effect for sensing, 80-nm SiO_2_ was electron-beam evaporated as the protection layer for electrodes only, while a 3-nm Al_2_O_3_ layer via atomic layer deposition (ALD) acts as the top-gate dielectric. Au nanoparticles (Au NPs) were then sputtering coated on the surface of Al_2_O_3_ as the anchoring sites for probes (Fig. 1c and Supplementary Fig. 1a–c). After probe conjugation, the Au NP – probe complexes act as the sensing gate, the surface charge of which can be altered after binding with the specific analytes. The sensor is thus chemically gated^31^ due to the electric field from the charged sensing gate. The electric field penetrates through the insulating Al_2_O_3_ layer and modulates the conductivity of the underlying channel as measured by the drain current at a constant drain voltage. The fabricated devices show the p-type transfer characteristics with Ohmic contact at the rGO-Au electrode interface (Supplementary Fig. 1d) after thermal annealing into reduced GO (rGO) (Supplementary Fig. 2). Moreover, most devices (~60% or ~30 of 50 in total) are closely distributed within the range of ~11 ± 1 kΩ and ~2 ± 0.2 (i.e., variation within ±10% with respect to the mode value) for the resistance and the on/off current ratio (Supplementary Fig. 1e, f), respectively. These pre-selected devices will be further screened by fault diagnosis, as described in the following section, where the selected devices will be subjected to sensor testing with L-cysteine, thioglycolic acid (TGA), and anti-*E. coli* antibody as the specific probes for detecting Pb^2+^, Hg^2+^ ions, and *E. coli* bacteria (Fig. 1d), respectively.

### Minimization of device variation and fault diagnosis

The sensing experiments reveal that FET sensors do not always exhibit a one-directional exponential response, which can be considered as ideal from the Langmuir’s theory of adsorption^32^. Instead, a bidirectional response could often be observed^33–36^ which may make it difficult to accurately predict the concentration of detected analytes. Therefore, it is crucial to identify and isolate the defective devices by correlating with non-ideal responses before testing in real water. To this end, developing a non-destructive testing (NDT) procedure (i.e., without damaging the actual sensing layer) will significantly advance the rapid screening of devices by obtaining knowledge about their behaviour a priori.

**Fig. 2:**
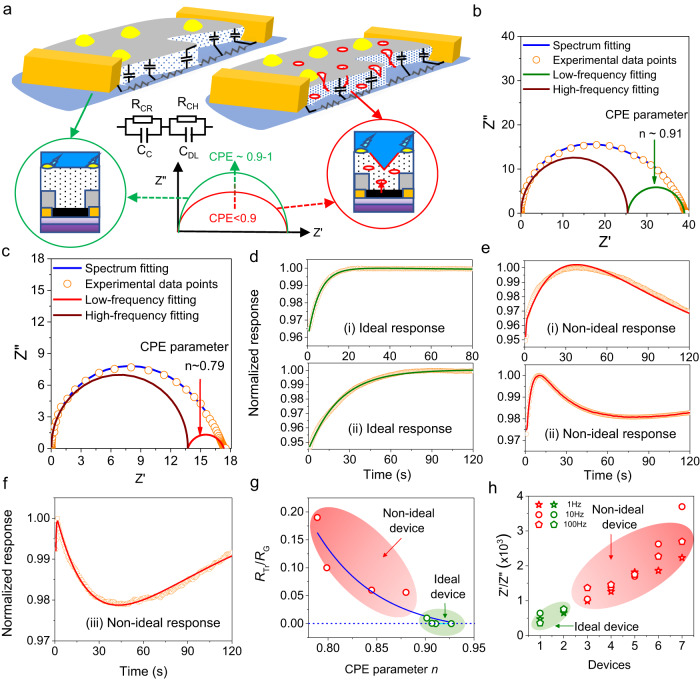
Alternating Current (AC) impedance spectroscopy for isolating non-ideal devices. **a** Schematic equivalent circuit model of channel resistance (R_CH_)-capacitance (C_DL_) pair and contact resistance (R_CR_) and capacitance (C_C_) pair of the ideal device and non-ideal device with defects (red circles) induced charge trapping from the channel due to the electric field from the gold-probe complexes (yellow circles). **b**, **c** Nyquist plots of impedance spectra fitted for the ideal device (ideal-like) and non-ideal device (with defects), respectively. Dots for experimental data points (yellow) and solid lines (brown, green and blue) for fitted curves. **d**–**f** The typical patterns of the response transient from Pb^2+^ ions exposure are shown in **d** for ideal and **e**, **f** for non-ideal devices, respectively. The response was relative to the baseline in air and normalized to the peak value in Pb^2+^ ion solutions. Background water was added prior to the Pb^2+^ ions exposure. The dotted (yellow circles) and solid lines (green and red) stand for experimental data points and fitted curves respectively. **g** Correlation of constant phase element (CPE) parameters by fitting the impedance spectra from the low-frequency semicircle with the magnitude of $\frac{{R}_{{Tr}}}{{R}_{G}}$ described in Eq. (1). **h** Clear separation of the ideal devices from non-ideal devices by the measured Zˊ/Z˝ ratio at low frequencies. The shaded areas (light red and green) in **g** and **h** show the respective region for non-ideal and ideal device category for calibration of the pre-selected sensors estimated from the non-destructive testing (NDT) procedure. The solid blue line (fitted) could be used for calibration of new devices under test.

Optical microscopy can be used to identify visible structural defects caused by fabrication, such as unsuccessful lift-off and discontinuous coating of the SiO_2_ protection layer (Supplementary Fig. 3a and Supplementary Fig. 3b). Despite being straightforward, such a method is tedious and thus inefficient when it comes to isolating the faulty devices during large-scale fabrication. More importantly, invisible defects (e.g., tiny cavities, Fig. 2a) are common, cannot be easily identified, and may adversely affect device performance, thus limiting the overall efficacy of optical microscopy as a pre-screening tool. For example, the film quality of the top gate dielectric layer (3 nm Al_2_O_3_) is key to our sensor performance. Upon adsorption of charged species, a perfect dielectric layer acts as a capacitor because the charges only accumulate on its surface. To characterize the quality of the dielectric layer, we developed an alternative approach by exploiting the wide-range and high-frequency (1 Hz – 5 MHz) impedance measurements. Specifically, this dielectric layer with capacitance C_DL_ is treated as a constant-phase element (CPE), which is in parallel connection with the channel material with resistance R_CH_. The electrical impendence of CPE at 1 Hz can be calculated as ${Z}_{CPE}=\frac{1}{2\pi } \big|{\tilde{Z}}_{CPE} \big|\exp (-n\pi i/2)$, where $n=1$ for an ideal capacitor and $n=0$ for a pure resistor. For a defective dielectric layer ($0 < n < 1$), its Nyquist plot will be squeezed from the semicircle (Fig. 2a).

Our pre-screening starts from the impedance measurements in deionized (DI) water. By considering the contact resistance R_CR_ and capacitance C_C_ at the interface between the channel material and source/drain electrodes, the equivalent circuit of our sensor structure is a serial connection of R_CH_ C_DL_ and R_CR_ C_C_. Since C_DL_ and C_C_ will have different values (typically C_DL_ > C_C_), there will be a supressed semicircle in the Nyquist plot which can be decomposed into two (quasi-)semicircles with C_DL_ (R_CH_) and C_C_ (R_CR_) lying in the low- and high-frequency regions, respectively. Figure 2b, c show the Nyquist plots for the ideal and non-ideal devices (i.e., without and with significant defects), where the ideal response is represented by a one-directional exponential (Fig. 2d) and the non-ideal response by a bi-directional exponential (Fig. 2e, f) when exposed to the Pb^2+^ solution. Impedance analyses clearly showed that the CPE parameter of the ideal-like devices ($n=0.91$) is larger than that of the defective devices ($n=0.79$). To identify the critical value that can separate the ideal responses from the non-ideal responses, the response transients are fitted with the superposition of two exponential functions that represent two competitive behaviors during sensing: the gating-induced current from the surface adsorption of Pb^2+^ ions and the defect-induced charge trapping from channel to the dielectric layer. The normalized response transient $R(t)$ could be introduced as

2 $$R\left(t\right)=[1-\exp \left(-\frac{t}{{\tau }_{G}}\right)]-\frac{{R}_{{Tr}}}{{R}_{G}}[1-\exp \left(-\frac{t}{{\tau }_{{Tr}}}\right)],$$

where ${R}_{G}$ and ${R}_{{Tr}}$ are the amplitudes of the gating and the charge-trapping-induced response with respective time constants ${\tau }_{G}$ and ${\tau }_{{Tr}}$. Using this equation, the normalized response transients were well fitted for both the ideal (Fig. 2d) and non-ideal (Fig. 2e, f) responses. To correlate ${R}_{{Tr}}/{R}_{G}$ with the quality of CPE, Fig. 2g shows the plot of CPE parameters (measured before sensing measurements) against ${R}_{{Tr}}/{R}_{G}$, which decays exponentially and should be negligible for ideal-like devices but is much larger for defective devices. For the given concentration of Pb^2+^ ions, the maximum normalized response is ~ 5%, as shown in Fig. 2d (ii). If one order of magnitude difference between the charge trapping and gating effects (i.e., ${R}_{{Tr}}/{R}_{G}=0.005$) is defined as the quantitative criterion to isolate the faulty devices, $n$ is extrapolated to be ~0.91 from the exponential fitting. This shows promising potential for predicting the behavior of devices prior to sensing (i.e., diagnosing the faulty devices) and can thus significantly minimize device variations. Note that the measurement of the leakage current between the source and the gate electrodes can also be used to characterize the quality of the top dielectric layer; however, this fails when either charge carriers in the channel or ions in the solution are only trapped without a diffusion path in the dielectric layer (see Supplementary Note 1). To expedite the procedure, we further calculated the Zˊ/Z˝ vs. frequency for various devices in the low-frequency region (1, 10, 100 Hz) and plotted for both types of devices (Fig. 2h). A clear transition was observed from the good to bad devices, suggesting that Zˊ/Z˝ < 1000 can be used as a criterion for ideal-like devices, thus eliminating the need for impedance spectra fitting. This electronic isolation of faulty devices is faster and accurate, and thus could be useful for large-scale device scanning and isolation. A comparison of the response distribution from the same concentration of toxins (5 ppb Pb^2+^) with and without device preselection is shown in Supplementary Fig. 4, and the range of the sensing response is much narrower after preselection.

### Noise spectral analysis on GFET under sensing environments

**Fig. 3:**
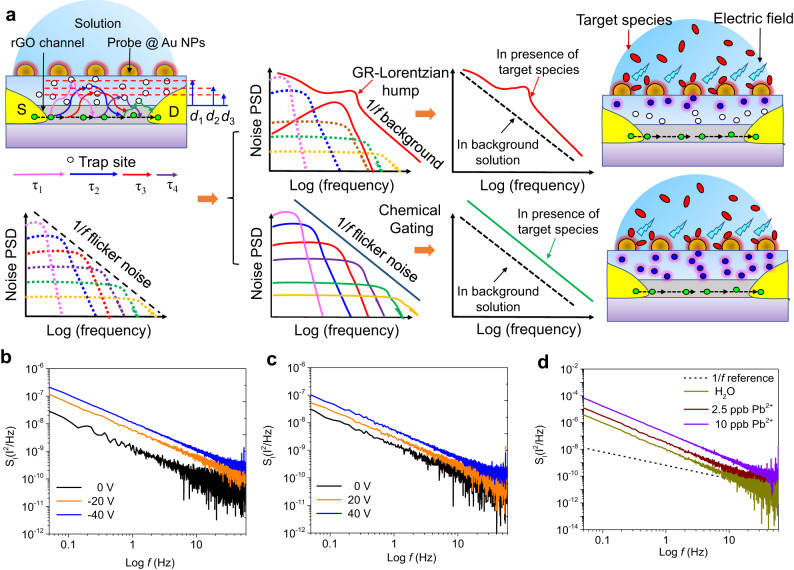
Noise spectral measurements of GFET sensors. **a** Schematic representation of the evolution of $1/f$ noise background due to the presence of defects at the channel-gate surface and target analytes. White circles in the upper left panel stand for defects in the top oxide layer, while the green circles in the upper left panel are charge carriers injected by electrodes into the channel. The blue spheres in the rightmost panels represent the specific defects responsible for the generation recombination (GR)-Lorentzian hump. Determined by its distance from its position (horizontal dashed lines) to the channel (d_1_, d_2_, d_3_, etc.) each defect has its unique characteristic time constants (*τ*_1_, *τ*_2_, *τ*_3_, etc. represented by colour arrows with different length) and unique power spectrum density (PSD) as shown in the dotted curves in PSD schematics with different colours. **b**, **c** Drain current noise PSD measured in air at negative (0 to −40 V) and positive (0–40 V) bottom gate bias, respectively. **d** Drain current noise PSD for clean water and Pb^2+^ ion solutions with different concentrations (2.5 and 10 ppb).

Due to its potential for integration with existing technologies and infrastructures for widespread diagnostic applications, the time-domain measurement of the total electronic response (e.g., drain current) in a FET sensor is commonly employed as the sensor signal for real-time monitoring of target analytes. However, its sensitivity and selectivity can be limited by the electronic noises, especially when the measured sensing signal is small (i.e., low signal-to-noise ratio at a low concentration of target analytes). Defects and surface states are intrinsic sources of noises which can cause fluctuations in charge carrier mobility and number. As a result, noise analysis in the sensor device can both provide a wealth of information about its fabrication quality and serve as a highly sensitive and non-destructive method for pre-screening. Complementary to the impedance studies above, the low-frequency drain current noise PSD is further adopted to characterize the quality of the top gate’s dielectric layer and its interface with sensor channel. As a generic phenomenon in FET-type electronic devices, generation-recombination (G-R) of the electronic charge carrier (i.e., trapping-detrapping) caused by defects in the gate oxide near the channel surface (the interface between Al_2_O_3_ and rGO in our sensor shown in Fig. 3a) will lead to fluctuations in the current. Determined by its distance from the channel (d_1_, d_2_, etc.), each defect has its own characteristic time constant $\tau$ and gives rise to a bulge in the shape of the Lorentz function, the envelope of which in a certain range can result in the 1/*f* noise (or flicker noise). When exposed to the sensing environment, the envelope evolution of the drain current noise PSD could generate either a Lorentzian hump over the 1/*f* background noise by a significant type of traps or simply a vertical shift by chemical gating (faulty vs. ideal-like devices, also see Supplementary Note 2)^37–40^.

To benchmark the quality of the top dielectric layer, the noise amplitude vs. the frequency of our GFET device was first measured at negative (0 to −40 V) and positive (0 to 40 V) bottom gate bias (Fig. 3b, c), respectively. Since noise in graphene is dominated by mobility fluctuation, Hooge’s empirical law^41,42^ is used to describe the drain current $1/f$ noise PSD (*S*_*I*_)

3 $${S}_{I}={N}_{A}\frac{{I}^{2}}{{f}^{\beta }},$$

where ${N}_{A}$ and I are the noise amplitude and mean current, respectively. $\beta$ is the fitting parameter, and deviation from its ideal value of 1 indicates the superposition of random telegraph signal (RTS) on the pure mobility fluctuation 1/*f* noise. The obtained value of *β* is ~1 for the bottom dielectric layer (300 nm-thick SiO_2_). We then conducted the noise measurements in water for various concentrations of Pb^2+^ ions (0-20 ppb). Due to the positive electric field, the increase in gate voltage at increasing lead-ion concentrations sequentially decreases the drain current level (Supplementary Fig. 5). The noise PSD of 1/*f* dependence with vertical shifts against concentration (Fig. 3d) indicates the chemical gating from the adsorption of Pb^2+^ ions, thus surface modification by various bio-chemical probes and antibodies will not essentially change any intrinsic behaviors of the devices in the sensor array structure. In addition, fitting the 1/*f* power spectrum in water yields a value of β ~ 2, suggesting the presence of RTS. Thus, defects in 3-nm Al_2_O_3_ across the rGO channel is inevitable and intrinsic from the ALD technique. However, the absence of G-R bulges implies that the as-deposited Al_2_O_3_ in the preselected devices from the impedance measurements functions as a decent dielectric layer.

To further validate this, the noise pattern against the gate bias is measured under both ambient and sensing environments. The measured noise in air shows a V-shaped pattern against the bottom gate bias with minimum around the charge neutrality point (Supplementary Fig. 6a). However, it changed into an M-shaped behavior when exposed to Pb^2+^ and *E. coli* (Supplementary Fig. 6b). Such transition into the M-shaped noise pattern might be due to the long-range scattering^43^ and spatial charge inhomogeneity^44^ from Pb^2+^ and *E. coli* adsorption on the sensor channel (discrete distribution of Au NPs as the top gate electrode vs. uniform SiO_2_/Si substrate as the bottom gate electrode). To quantify the gate bias exerted from the surface adsorption, Grahame’s Eq. (4) is adopted to approximate its magnitude around 298 K, as shown below:

4 $$\varphi=\frac{2{k}_{B}T}{e}{\sinh }^{-1}\left(\frac{e\sigma {\lambda }_{D}}{2\varepsilon {\varepsilon }_{0}{k}_{B}T}\right),$$

5 $${\lambda }_{D}=\sqrt{\frac{\varepsilon {\varepsilon }_{0}{k}_{B}T}{{e}^{2}{\sum }_{i}{Z}_{i}^{2}{C}_{i}\left(M\right)}},$$

where the surface potential $\varphi$ is governed by the charge density $\sigma$ and Debye length ${\lambda }_{D}$, which depends on the ionic concentration $Ci$ (in Molar) and its valence charge *Z*_*i*_ in tap water. $\varepsilon ({\varepsilon }_{0})$ is the relative dielectric constant in water (vacuum permittivity), while ${k}_{B}$ and $T$ are the Boltzmann constant and temperature in K, respectively. ${\lambda }_{D}$ in Eq. (5) for our tap water sample is ~ 4.5 nm when only the major minerals are considered (see Methods). The estimated maximum molecular probe density^45^ of the closely self-assembled, monolyer L-cysteine (negatively charged) is ~−6e nm^−2^; $\varphi$ in Eq. (4) can then be calculated as ~−285 mV, which is equivalent to a bottom gate bias of −26.3 V (dielectric constant of thin ALD Al_2_O_3_ film (~3.6)^46^ is comparable to that of 300-nm thick SiO_2_ (3.9)). This sets the upper limit of change in the top gate bias from the surface adsorption of lead as its concentration increases. The estimated top gate bias and measured noise pattern suggest that the pre-screened sensor devices are reliable for the subsequent measurements in flowing tap water.

### Sensing measurements of toxins in flowing tap water

**Fig. 4:**
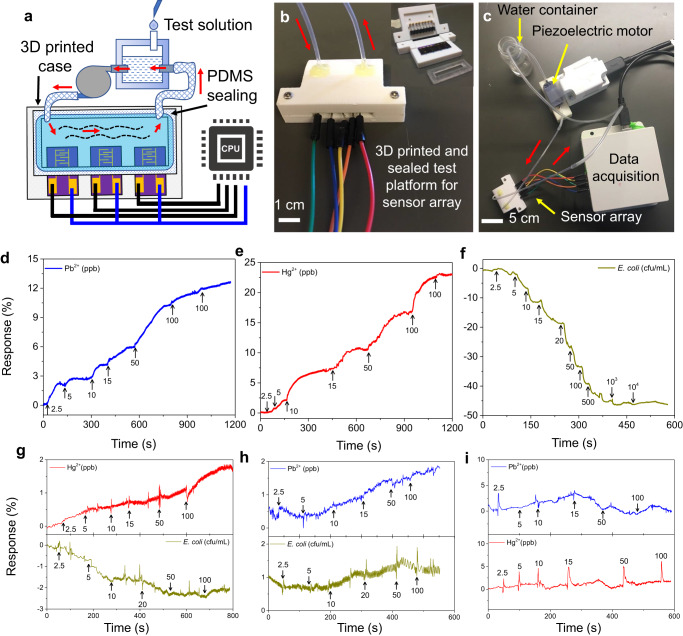
Measurements of a GFET sensor array in a flowing water system. **a** Schematic experimental setup for continuous sensing. The sensor array housed in the chamber is sealed with a polydimethylsiloxane (PDMS) mold from the source-drain contact. Water is drawn by the piezoelectric motor into the sensor chamber and then continuously flows back to the external container to mimic the water flow. **b** Photograph of a 3D-printed sealed chamber with the sensors embedded inside. Inset is the cell interior. **c** Photograph of a piezoelectric micromotor and an external water container connected to the sealed sensor chamber. **d**–**f** Response transient patterns for Sensor 1 (Pb^2+^), Sensor 2 (Hg^2+^), and Sensor 3 (*E. coli*), respectively. **g**–**i** Selectivity studies for Sensor 1 (Hg^2+^ and *E. coli*), Sensor 2 (Pb^2+^ and *E. coli*), and Sensor 3 (Pb^2+^ and Hg^2+^), respectively. The arrows signify when the target toxin solutions were added.

To enable continuous sensing in flowing water, the GFET sensor array with three individual sensor chips is housed in a 3D-printed closed-loop chamber, which is powered by a piezoelectric pump to allow the water flow between it and the external container through the inlet and outlet vessels (Fig. 4a–c). Prior to the sensor tests, it should be noted that the performance of a FET water sensor can be affected by other factors as well, especially the pH (Supplementary Fig. 7) and the ionic strength of the sample solution. At the detection level (ppb) for the heavy-metal ions of interest here, both pH and ionic strength can be considered as constants for the tested tap water (pH~ 7.7 and Debye length of ~4.5 nm) since they are dominated by the stray ions at much higher concentrations (e.g., Na, Mg, Ca at ppm level). Moreover, the sizes of the selected molecular probes (~ sub-nm) are smaller than the Debye length and electric fields from the surface-adsorbed heavy-metal ions will eventually cause the resistance change in the sensor channel.

During the sensing test at room temperature, water droplets containing heavy metals and *E. coli* bacteria at different concentrations were continuously injected into the external container with clean tap water as the background. By recording the resistance change of the sensor channel with time, the presence of heavy metals and *E. coli* bacteria can be both identified and quantified. Figure 4d–f shows the typical response transients from Sensors 1-3 in the sensor array for Pb^2+^, Hg^2+^ ions, and *E. coli* bacteria, from which we can see the stepwise increase and decrease of the sensor response (i.e., resistance) for heavy-metal ions and *E. coli* bacteria. This can be expected for the p-type semiconducting channel of rGO, since heavy-metal ions are positively charged and *E. coli* bacteria are negatively charged (zeta potential from −4.9 to −33.9 mV^47^) at the pH of tap water (~7.4). The sensor response is up to ~10% for Pb^2+^ and ~16% for Hg^2+^ ions at 100 ppb, while down to ~−45% at 10^4^ cfu/mL. In addition, the sensor response can be distinguished clearly from the background at the concentration of 2.5-5 ppb and cfu/mL for heavy-metal ions and *E. coli* bacteria, respectively. Considering the dilution effect of the background water, the effective limit of detection can be lower. To further confirm that the sensor response is due to the presence of detected toxins, the cyclic response and sensor recovery were tested by alternatively injecting the toxin solution and the clean tap water. Indeed, opposite sensor responses were observed, though desorption of toxins from the sensor surface (i.e., sensor recovery) is a relatively slower process than their adsorption on the sensor surface (Supplementary Fig. 8).

The selectivity study for Sensors 1-3 (Fig. 4g–i) revealed minimal cross-sensitivity among the sensors. The unidirectional exponential response transients for each specific device ensured there was no charge carrier leakage from the channel into the top gate oxide or the secondary contribution of current during measurement as the concentration increased. The specific affinity towards heavy metals with cysteine, glutathione, and TGA can also be found elsewhere^15,48,49^. Other bacteria (with the non-pathogenic *E. coli* strain DH5a and the plant-pathogenic bacterium *Dickeya dadantii 3937*) present in flowing water with Sensors 1-3 (Supplementary Fig. 9) showed a negligible interaction with all devices. It is noted that some cross-sensitivity was observed in Sensor 1 (targeted for Pb^2+^) in higher concentrations of Hg^2+^ ions. This can be compensated by the machine learning (ML) model, as discussed later. Besides, the much lower sensor response (within ~+/−2%) compared to the sensitivity tests in Fig. 4d–f (from ~−45% to 16%) during the testing period (e.g., 500−800 s) suggest that the drifting of the background signal can be reasonably neglected.

### Machine-learning-assisted classification and quantification

**Fig. 5:**
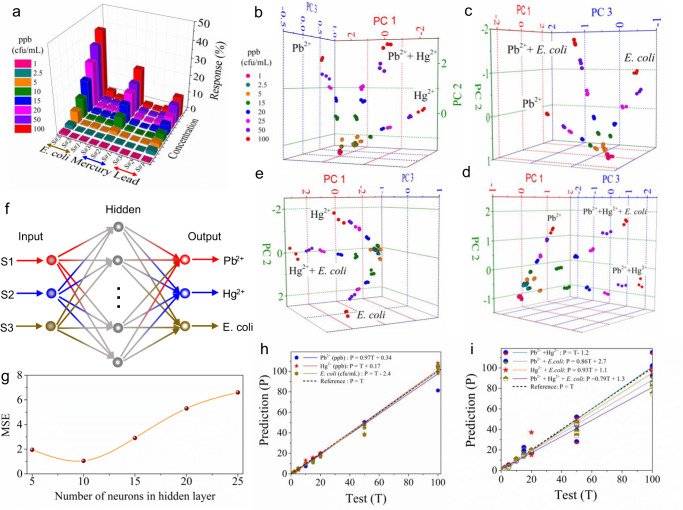
Machine learning (ML) modeling for classifying and quantifying various mixed ionic and bacterial species in flowing water. **a** Summary of responses from Sensors 1-3 for Pb^2+^, Hg^2+^, and *E. coli* shown in Fig. 4d–i. **b**–**e** Principal component analysis (PCA) plots for the classifications of Pb^2+^, Hg^2+^, *E. coli*, and their mixtures. **f**, **g** Schematic of a two-layer artificial neural network (ANN) used for training and mean squared error (MSE) values for various numbers of neurons in the hidden layers. **h**, **i** Plots of the test and the predicted concentrations for Pb^2+^, Hg^2+^, and *E. coli* (ppb or cfu/mL) and their mixtures from the trained ANN model. Here, the dash line (Y = T) represents the perfect match between the test and the predicted concentrations for unknown samples while the solid line represents the fitting of the target concentrations predicted from the trained ANN model.

The sensing performance of a single sensor is typically calibrated by revealing the one-to-one relationship between the target concentration and the sensing response. While predictions from such calibration could be accurate for a single type of analyte, it can fail with the presence of other types of interference analytes, especially at much higher concentrations. For example, as summarized in Fig. 5a, for the sensor response of a single type of analyte only, Pb^2+^ (Hg^2+^) ions at low concentrations will not be differentiated from Hg^2+^ (Pb^2+^) ions at a much higher concentration simply by the sensing response from a single device. This issue also cannot be addressed by processing the sensing data from multiple devices independently (e.g., calibrating Pb^2+^, Hg^2+^, and *E. coli* individually from the sensor array). Here, we selected the time-domain response output acquired at the same time for each individual device in the sensor array for ML-assisted classification and quantification in various mixture conditions. Using the concurrent multi-sensor responses as inputs after exposing the sensor array to various combinations of toxins (mixed ions and bacteria), principal component analysis (PCA) successfully classified and quantified the target components (Fig. 5b–e). Despite some overlap among the target species in the low-concentration region (<2.5 ppb or cfu/mL), they are well below the corresponding thresholds for tap water standards regulated by the World Health Organization^50,51^. Hence, it would still be highly significant to obtain an early-warning signature if quantified properly. To do so, the sensor array data was further analyzed using a two-layer feedforward artificial neural network (ANN) as shown in Fig. 5f, which was tested for various numbers of neurons in the hidden layer, with the minimum mean squared error (MSE) at 10 neurons (Fig. 5g). Indeed, the predicted outputs from the trained ANN model show the successful quantification of each individual toxic element in their various mixtures (Fig. 5h, i).

## Discussion

The sensing performance of a FET sensor is governed by its transfer characteristics which are intrinsic to the channel material. The statistical values of the electronic properties in the rGO channel from the wet transfer technique can be further tailored by the fabrication and annealing conditions. While a CPE parameter *n* of > 0.91 could be a generic quantitative criterion for FET sensors with an ideal-like top dielectric layer (i.e., independent of its thickness and type), the identified ratio of Zˊ/Z˝ < 1000 in the low-frequency range (1-100 Hz) can be specific to the channel material and sensor architectures (e.g., its lateral size, contact resistance). The unique value of Zˊ/Z˝ for other materials and sensor structures can be straightforwardly obtained by repeating the procedures of impedance measurements, as described in Fig. 2. The low-frequency noise measurement will not only signify the presence (G-R bulge) and absence (vertical shift or chemical gating) of a significant type of defect trap over the 1/*f* noise background, but also show a much higher sensor response (Supplementary Fig. 10), thereby providing opportunities either in ultralow detection whenever necessary or sensing in harsh environments when the sensor response can be dampened (e.g., by much higher ionic strength and the presence of interference substances in other types of water sources like rivers, lakes, or wastewater). The detection and expansion of other types of emerging toxins (e.g., pharmaceuticals, pesticides, per- and polyfluoroalkyl substances) in water can be further realized by simply replacing the specific probes and increasing the number of individual devices in the sensor array, while the predication accuracy can be recalibrated by optimizing the number of neurons in the hidden layer and/or in the hidden layer itself when training the ML model for classification and quantification. The reversibility and reproducibility of the sensors are central to their practical applications. Depending on the binding kinetics between the target analyte and its specific probe, the time scale for a sensor to return to its initial state will vary. While fast recovery is often preferred, reversible sensing can be achieved by either selecting and designing specific probes with moderate binding strength or by applying an external bias to accelerate surface detachment through the electrostatic repulsion. Finally, considering that the property of tap water from different sources can vary in a wide range (e.g., pH, hardness, etc.) due to the regional regulation, the real field applications are promising when a more sophisticated ML model can be constructed to incorporate more relevant parameters such as pH, ionic strength, and environmental temperature for a more accurate prediction.

In summary, we demonstrated a scalable approach for developing GFET sensor array devices functionalized with multiple biochemical ligands and antibodies as specific probes for detecting heavy metals and *E. coli* bacteria simultaneously in running tap water. The wafer-scale deposition of GO dispersion by spin-coating on the silicon substrate provides narrow distributions of both the resistance and current on/off ratio, which are highly desirable in electronic sensors for commercialization. To eliminate the sensors with non-ideal sensing kinetics, ideal-like devices could be pre-selected by the high-frequency impedance and low-frequency noise measurements. With the aid of ML, multiple toxins in real tap water flow can be successfully identified and quantified with a high accuracy. Our strategies for the scaled-up manufacturing of FET sensors and the minimization of device variations hold promise for future dynamic prediction of various toxic substances in tap water in real time for water risk management.

## Methods

### Large-scale nanofabrication of GFET sensors

Customized monolayer (99%) GO dispersion in water with a lateral flake size of 5-10 μm (GaoxiTech, 10 mg/mL) was spin-coated onto the silicon wafer with a 300-nm SiO_2_ layer, which was pre-treated with oxygen plasma to enhance its surface hydrophilicity. Then, 20 mL GO dispersion, which was diluted 16 times, was first placed on the wafer for 2 s and then spin-coated at 1500 revolutions per minute (rpm) for 30 s such that individual GO flakes were uniformly distributed on the wafer surface. Two steps of lithography were used to fabricate the sensor device. The gold (Au) electrodes were first deposited into the interdigitated pattern (a finger width of 2 μm, an inter-finger spacing of 5 μm, and a thickness of 50 nm) defined using a Maskless Aligner (MLA150). After that, an 80 nm-thick SiO_2_ layer was thermally evaporated to conformally encapsulate the interdigitated Au electrodes by masking the GO surface using a photoresist. Due to the atomic thickness, the developer solution might have some impact on electronic properties of the rGO (e.g., scattering the charge carrier and degrading its mobility). The SiO_2_ passivation layers can exclude solution-induced interference to the device from the contact pad (rGO-Au interface) with only the sensing region accessible for analytes. Next, the wafer was diced into individual sensors using a circular diamond cutter and then washed with ethanol to remove the photoresist. The diced sensors were kept in an ALD chamber (200 °C) to deposit the top gate oxide (3-nm Al_2_O_3_) on the sensor surface. The top gate acts as the surface passivation to prevent direct charge transfer to the channel from the test solution. Finally, Au nanoparticles (Au NPs) were uniformly deposited onto the device using a sputter-coater instrument (Quorum Q300T, 3 s pulse duration at 10 mA).

### Morphological and electronic characterizations

An S-4800 UHR Hitachi field-emission scanning electron microscope (SEM) was used to characterize the surface morphology of the sensor device at an acceleration voltage of 10 kV. The atomic force microscopy (AFM) characterization was conducted with a 5420 AFM from Agilent Technology (Cantilever PPP-NCH) in ACAFM mode. Raman spectroscopy was performed with a Renishaw Raman spectrometer (1000B). X-Ray photoelectron spectroscopy (XPS, HP 5950 A with Mg Kα) was used to characterize the surface chemistry of GO before and after thermal annealing. The output and transfer characteristics of the FET sensors were measured by the Keithley 4200 semiconductor characterization system at ambient temperature. The impedance analyses were performed using the Ametek Scientific Instruments (PARSTAT 4000 A) with a frequency ranging from 1 Hz to 5 MHz by connecting to the source-drain terminals of GFET devices.

### Sensor surface functionalization with various ligands

After sputter-coating, the individual GO/Al_2_O_3_/Au NPs sensors were thermally reduced to rGO/Al_2_O_3_/Au NPs at 400 °C for 1 h in an Argon environment. The GFET sensor array was then modified with specific probes. For Sensor 1, the saturated L-cysteine solution (∼5 µl) was dropped onto its active surface inside a closed chamber for 1 h. Sensor 2 was functionalized by immersing it into 10 mM TGA solution. Both L-cysteine and TGA have the thiol group (-SH) that creates a link to the Au NPs via the Au-S bonding. Sensors 1 and 2 were washed with deionized (DI) water and dried with dry N_2_ gas. Sensor 3 was immersed in an aminoethanethiol (AET) solution with a concentration of 10 mM for 1 h. After thoroughly rinsing with DI water and drying under a stream of nitrogen gas, the modified device was treated with a 25% glutaraldehyde (GA) solution at 25 °C for 1 h. The device was then incubated in the phosphate-buffered saline (PBS) with anti-*E. coli* O157:H7 (10 mg mL^−1^) antibodies at 4 °C for 12 h. Finally, the device was incubated with a blocking buffer (0.1% Tween 20) for 2 h at 25 °C and then washed with PBS.

### Water sample preparation and characterizations

Mercury (II) chloride and lead (II) nitrate were used to prepare the Hg^2+^ and Pb^2+^ ion solutions in tap water collected in the lab, pH of which was measured by the OHAUS’ Aquasearcher™ AB33PH-B bench meter. The pH of collected tap water increased from 7.46 to a steady-state value of 7.67 after exposure to CO_2_ from air. The concentrations of prepared metal ion solutions were quantified by the inductively coupled plasma - mass spectrometry (ICP-MS) with an error <±5%. To characterize the ionic strength of the background tap water, the major mineral species were also quantified by the ICP-MS that include Na^+^ (0.4 mM), Mg^2+^ (0.5 mM), Ca^2+^ (0.8 mM), Cl^-^ (0.4 mM), and SO_4_^2-^ (0.2 mM). When calculating the Debye length in Eqs. (5), 2.2 mM bicarbonate (HCO_3_^-^) was used to meet the charge neutrality as it is the dominant form (~99%) of carbon species over carbonate (CO_3_^2-^) in water at pH <8. The bacteria samples were obtained from the *E. coli* cell culture (O157:H7 cells), and the non-pathogenic *E. coli* strain DH5a and the plant-pathogenic bacterium *Dickeya dadantii* 3937 were used to check the cross-sensitivity. *E. coli* of 10^6^ cfu/mL in 1x PBS was used as the source concentration and then diluted in tap water to specific low concentrations.

### Testing the GFET sensor array in a 3D-printed chamber

The sensing tests were conducted in a 3D-printed test chamber with a diced PDMS micro-reservoir integrated on the sensor’s active area. All sensor devices were kept in a predefined case, with the bottom of the case matching the dimensions of the sensor. The top part of the case integrated with the PDMS mold matched on the bottom part. An inlet and an outlet system were incorporated using a USB-powered piezoelectric motor. The top and bottom of the case were clipped to prevent water leakage. All the sensors were in contact with the flowing water as it moved through the chamber. The motor ran continuously to maintain an even flow of water over the sensors. During the sensing tests, we started with 2 mL background tap water in the external container that was connected to the sensor chamber by the inlet and outlet vessels and then added each concentration sequentially at a volume of 2 mL. An Arduino microprocessor controller with an external analog-to-digital converter (16-bit, ADS1115) was used to connect the sensor array to the HyperTerminal software for multi-channel data acquisition and data storage. The controller was programmed to quickly scan three devices consecutively in multiplexing mode. The noise analysis was done separately with a Keithley semiconductor analyzer in a high-speed data acquisition mode (sampling frequency of ~200 Hz). After the sensor reached equilibrium, a current of 1–5 s was collected for fast Fourier transform analysis. The sensor response (${S}_{R}=\frac{\Delta R}{{R}_{0}}*100\%$) was defined as the relative change of channel resistance ($\Delta R=R-{R}_{0}$) upon exposure to the target analytes normalized to its initial value (${R}_{0}$). ML analyses were performed in MATLAB with ANN trained through the Levenberg-Marquardt algorithm. About 30 sensors were preselected from a batch (50 devices) by resistance and drain-current on/off ratio and further down to ~20 by NDT-based measurements. Six GFET sensor arrays were finally tested against heavy-metal ions, *E. coli* bacteria, and their combinations in tap water.

### Reporting summary

Further information on research design is available in the Nature Portfolio Reporting Summary linked to this article.

## Supplementary information

Supplementary Information

Reporting Summary

## Data Availability

Relevant data supporting the key findings of this study are available within the article and the Supplementary Information file. All raw data generated during the current study are available from the corresponding authors upon request.
