# Supplementary material for: Scalable graphene sensor array for real-time toxins monitoring in flowing water
Source: Nat Commun. 2023 Jul 13;14:4184. doi: 10.1038/s41467-023-39701-0 (PMC10345017; doi:10.1038/s41467-023-39701-0)
Supplement: Supplementary file 1 — Supplementary Information [file 41467_2023_39701_MOESM1_ESM.pdf]

Scalable graphene sensor array for real-time toxins monitoring in flowing water

Arnab Maity,[1†] Haihui Pu,[1,2,3†] Xiaoyu Sui,[1,2,3] Jingbo Chang,[1] Kai J. Bottum,[1] Bing Jin,[1] Guihua Zhou,[1] Yale Wang,[1] Ganhua Lu,[1] Junhong Chen[1,2,3]*

[1] Department of Mechanical Engineering, College of Engineering & Applied Science, University of Wisconsin-Milwaukee, Milwaukee, WI 53211, USA.

[2] Pritzker School of Molecular Engineering, University of Chicago, Chicago, IL 60637, USA. E-mail: junhongchen@uchicago.edu

[3] Chemical Sciences and Engineering Division, Physical Sciences and Engineering Directorate, Argonne National Laboratory, 9700 S. Cass Ave., Lemont, IL 60439, USA.

†: These authors contributed equally to this work.

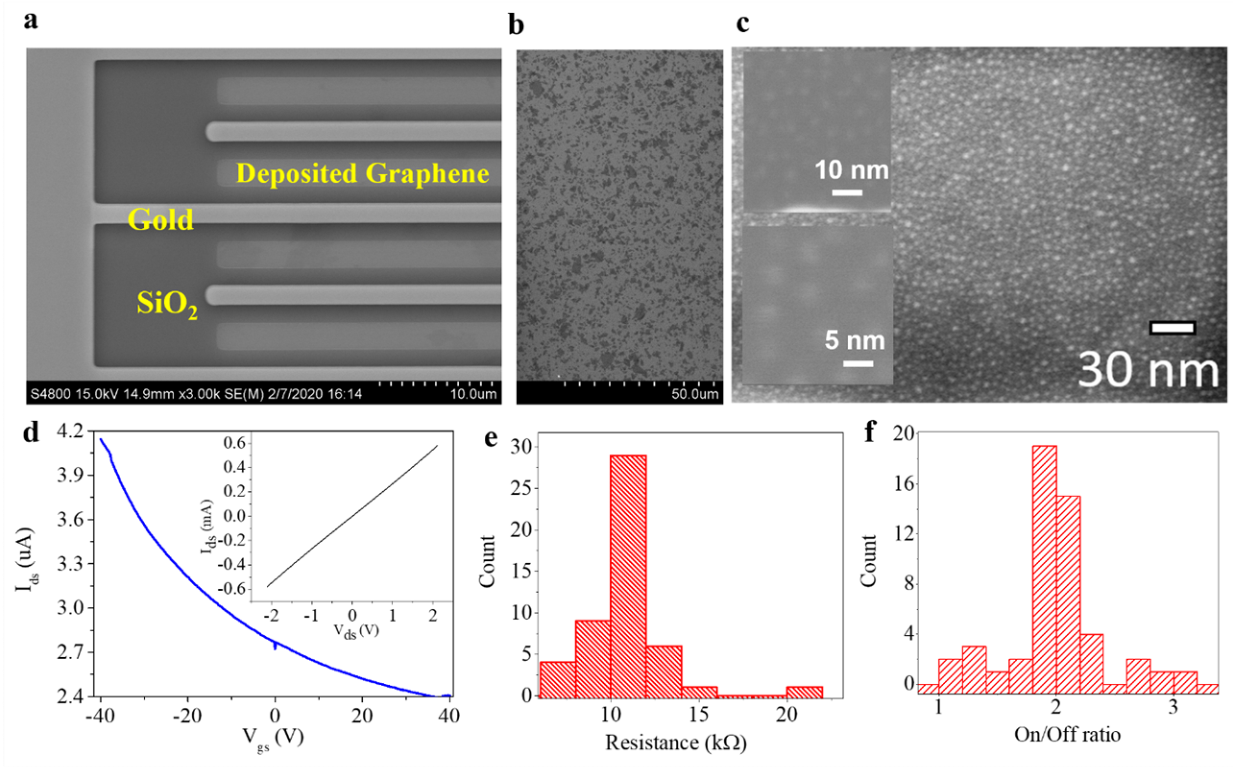

Supplementary Figure 1 | Characterization of the GFET device, measured transfer properties, statistical distribution. SEM images of (**a**) SiO_2-protected GFET device, (**b**) GO layers, and (**c**) sputtered Au NPs distribution on an ALD Al_2O_3 surface with the insets being magnified local views. **d**. Typical transfer characteristics for V_{gs} (-40 V to +40 V) and V_{ds}= 0.01 V with I_{ds}-V_{ds} output shown in the inset. **e-f.** Resistance and drain current on/off ratio distributions of fabricated sensor devices. Here, the on/off ratio is defined as the ratio of drain current at V_{gs}= -40 V over V_{gs} = 40V.

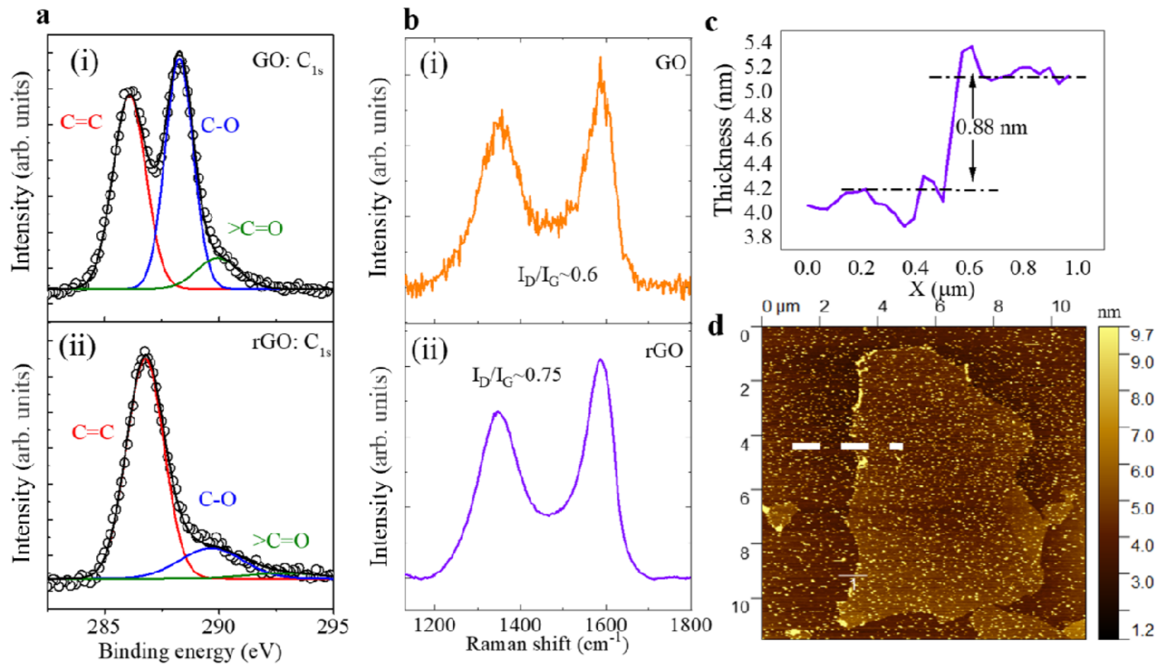

Supplementary Figure 2 | Characterization of the spin-coated GO. a XPS studies in GO (i) and rGO (ii) for C_{1s} position with decreased surface functional groups (i.e., recovery of sp^2 carbons) after thermal annealing; **b** Raman spectra of GO (i) and rGO (ii) with the large value of the I_D/I_G ratio implying increased structural defects induced by the thermal removal of surface oxygen groups. **c-d** AFM thickness profile of the spin-coated GO flake, which is nearly double that of a single graphene layer due to the surface functional groups.

a **b**

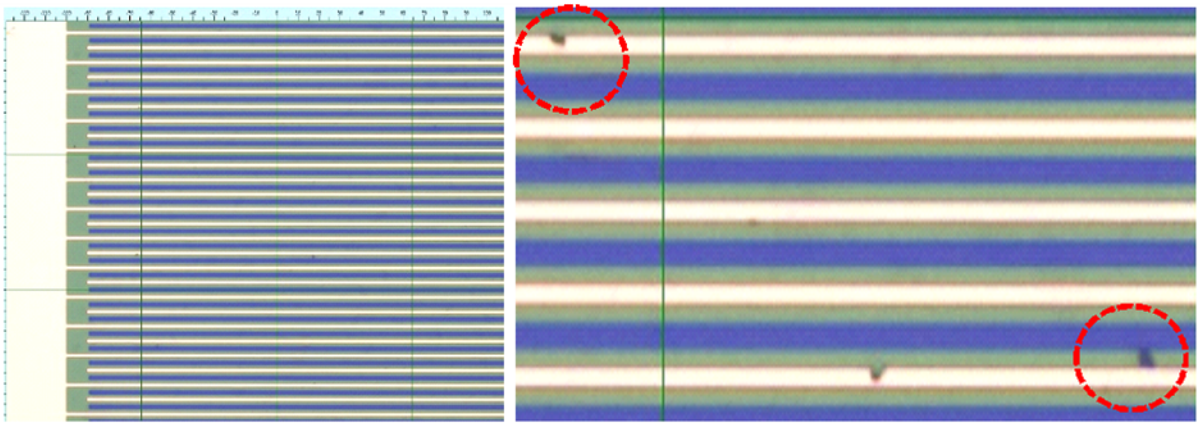

Supplementary Figure 3 | Microscopic images of interdigitated electrodes protected with an SiO_2 layer. The optical image comparison of device (**a**) without and (**b**) with defects.

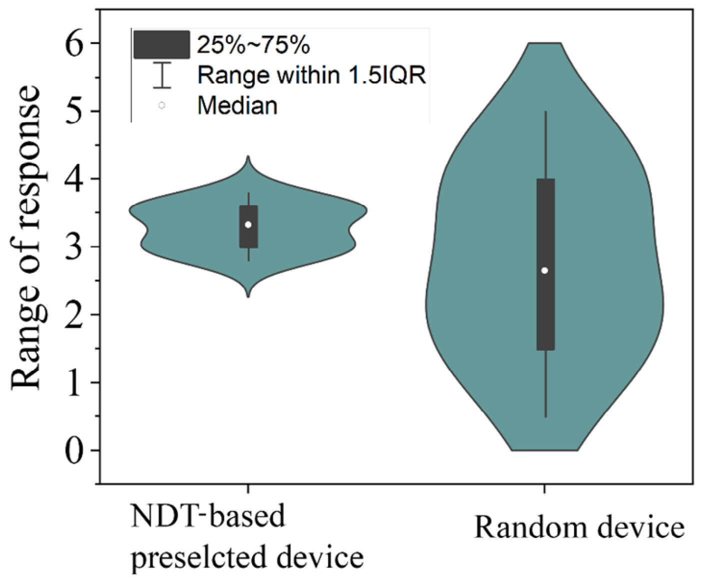

Supplementary Figure 4 | Comparison of the response distribution from the device variation between the NDT-selected-based device and the randomly selected device. Comparison of the response distribution from the same concentration of toxins (5 ppb Pb^{2+} ion) with and without device preselection (ten devices for each case). The NDT-based devices were selected using the criteria shown in the light green shaded region of Fig. 2 g-h. Clearly, the preselected sensor devices show a much narrower interquartile range (IQR) of sensing response.

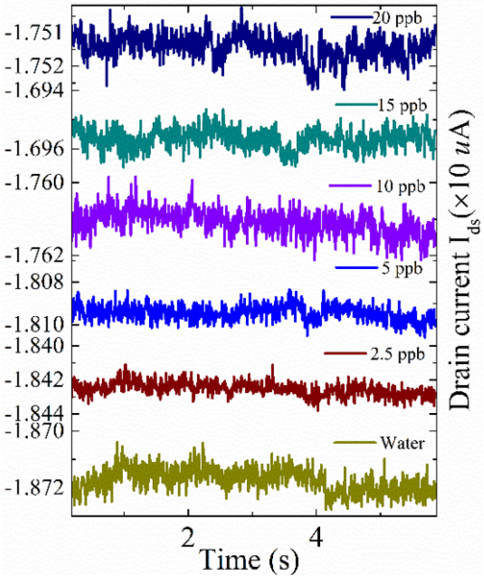

Supplementary Figure 5 | Noise measurement in the time domain for various concentrations of Pb^{2+} ions.

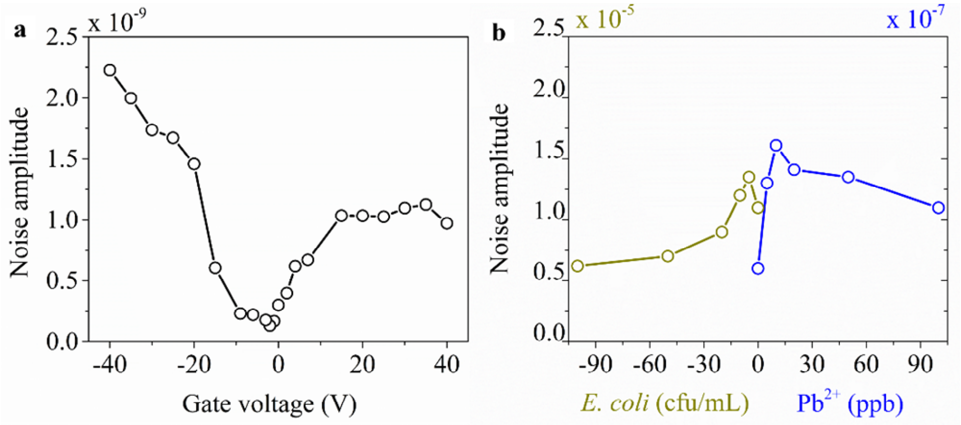

Supplementary Figure 6 | Noise amplitudes of sensor device measured at 10 Hz against bias voltage from (**a**) the bottom-gate oxide in air and (**b**) the top-dielectric layer in water with the presence of Pb^{2+} ions and *E. coli*.

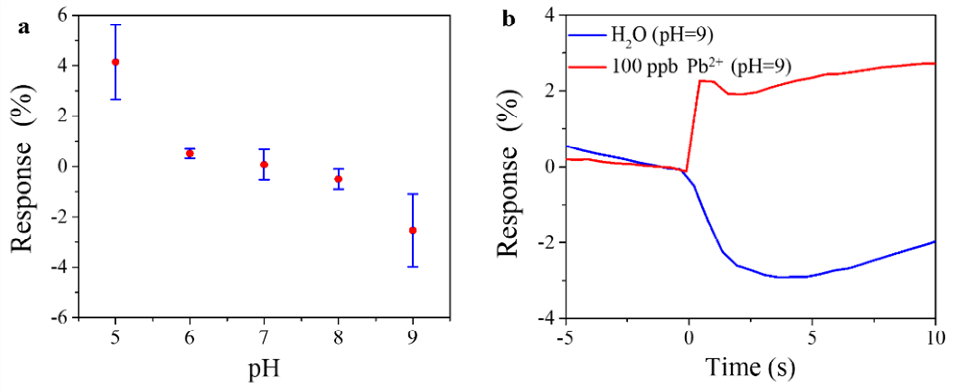

Supplementary Figure 7 | Effect of pH on sensor response. a. Sensing response attributed to pH from 5 to 9. **b**. Sensing responses to clean water and lead solution with the same pH at 9.

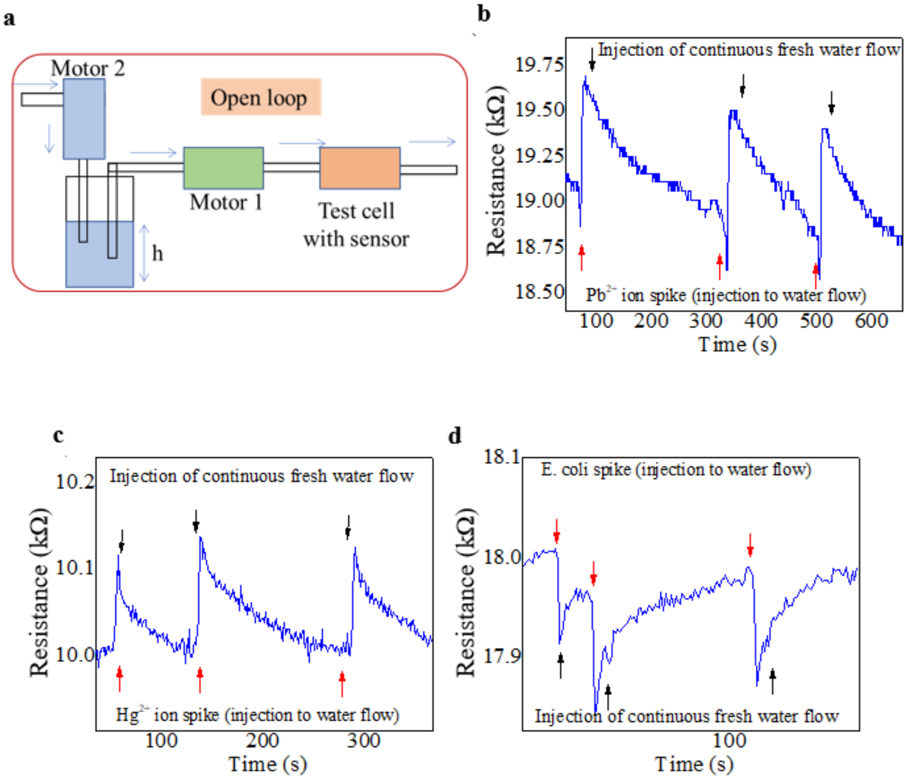

Supplementary Figure 8 | Cyclic response and sensor recovery by washing in a flowing water system. a. Schematic setup of controlling water flow during the washing processes. The typical cyclic performance during toxin spike and subsequent recovery in continuous tap water flow for (**b**) Pb^{2+}, (**c**) Hg^{2+}, and (**d**) *E. coli*.

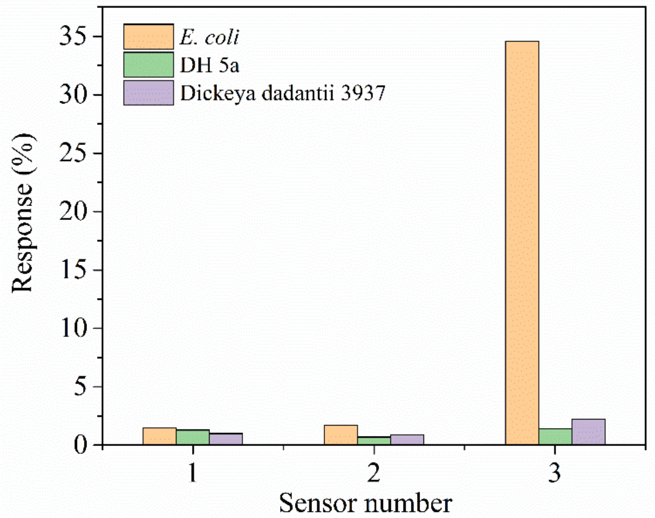

Supplementary Figure 9 | Sensor response to other common bacteria. The response comparison for *E. coli* with the non-pathogenic *E. coli* strain DH5a and the plant-pathogenic bacterium *Dickeya dadantii* 3937) in Sensors 1-3 in the GFET sensor array.

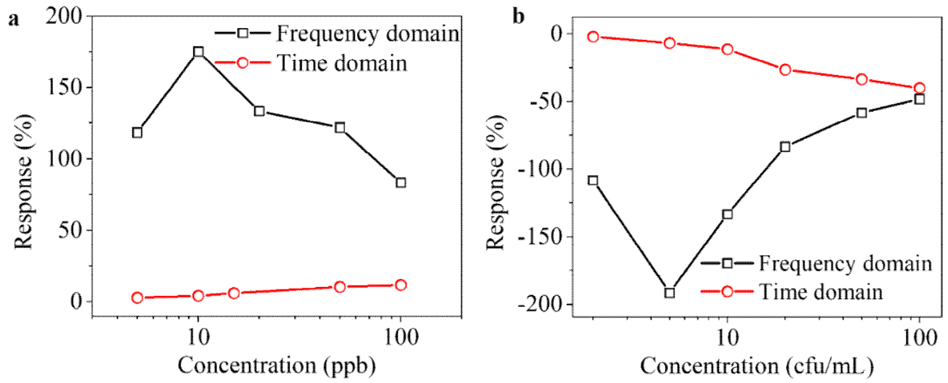

Supplementary Figure 10 | Comparison of sensor response in the frequency and time domains. a Pb^{2+} ions, and **b** *E. coli*.

Supplementary Note 1. Trapping/de-trapping vs. diffusion of charge carriers and ions in the dielectric layer

A perfect dielectric layer can act as an ideal capacitor with a constant specific capacitance with respect to thickness (or its dielectric constant will not change). The quality of a dielectric layer depends on the types of defects and how they are formed and distributed. For continuous cracks, ions in an aqueous environment and charge carriers in the channel can diffuse and migrate through the defective dielectric layer driven by the external electric field. As a result, the leakage current can be measured to characterize the quality of a dielectric layer in this scenario as well. However, when only discrete tiny cavities are present, either charge carriers from the channel or ions in the solution will be only trapped inside these cavities by the electric field and no current will flow. These trapped charge carriers and ions in the dielectric layer can modulate its dielectric constant (i.e., capacitance), the change of which suggests that it is not an ideal capacitor and thus can be characterized by the CPE parameter n. As a result, these cavities will result in the bidirectional sensing response. For example, as shown in Fig. 2g and f, the surface adsorption of Pb^{2+} ions can increase the electron concentration in the p-type rGO channel due to the gating effect, leading to the drain current decrease or resistance increase. However, electrons can migrate from rGO channel and be trapped into the cavities in Al_2O_3 layer with the electric field of adsorbed Pb^{2+} ions. Thus, the gating effect induced increase of electron concentration in rGO starts to decrease, which gives rise to the opposite direction of sensing response.

Supplementary Note 2. Trap influence in the shape evolution of noise spectral measurements

Generally, one single trap is associated with a unique relaxation time (or frequency) and its power spectrum signifies a Lorentzian type. However, for widely distributed traps, each individual relaxation frequency superimposes and exhibits the $1/f$ type power spectrum. This $1/f$ power spectrum could act as background in a 2D materials-based FET sensor. The shape of the power spectrum transits to a Lorentzian type when some biomolecules are attached or when gases are adsorbed directly to the surface of the channel material. The appearance of the specific Lorentzian hump over the $1/f$ background is associated with a certain corner frequency (f_c), which has been used to selectively differentiate the testing species[1-4]. However, a vertical shift of the $1/f$ spectrum in the presence of target species could also arise due to the gate-induced charge carrier modulation inside the channel with the presence of high-quality gate oxide, preventing the direct contact between the sensor channel and target analyte[2]. As a result, the flicker noise (associated with the gating effect due to probe-species binding) is dominant over the random telegraphic signals (related to the binding-unbinding of target species at the channel interface).

Supplementary References:

1. Sergey Rumyantsev, Guanxiong Liu, Michael S. Shur, Radislav A. Potyrailo, and Alexander A. Balandin. Selective Gas Sensing with a Single Pristine Graphene Transistor. *Nano Lett* **12,** 2294 (2012).
2. Qiushi Guo, Tao Kong, Ruigong Su, Qi Zhang, and Guosheng Cheng. Noise spectroscopy as an equilibrium analysis tool for highly sensitive electrical biosensing. *Appl Phy Lett* **101,** 093704 (2012).
3. Gengfeng Zheng, Xuan P. A. Gao, and Charles M. Lieber. Frequency Domain Detection of Biomolecules Using Silicon Nanowire Biosensors. *Nano Lett* **10,** 3179 (2010).
4. Jing Li, Sergii Pud, Michail Petrychuk, Andreas Offenhäusser, and Svetlana Vitusevich. Sensitivity Enhancement of Si Nanowire Field Effect Transistor Biosensors Using Single Trap Phenomena. *Nano Lett* **14,** 3504 (2014).
