# Supplementary material for: Scalable graphene sensor array for real-time toxins monitoring in flowing water
Source: Nat Commun. 2023 Jul 13;14:4184. doi: 10.1038/s41467-023-39701-0 (PMC10345017; doi:10.1038/s41467-023-39701-0)
Supplement: Supplementary file 2 — Reporting Summary [file 41467_2023_39701_MOESM2_ESM.pdf]

Corresponding author(s): Junhong Chen

Last updated by author(s): Jun 12, 2023

Editorial Policy Checklist

This form is used to ensure compliance with Nature Portfolio editorial policies related to research ethics and reproducibility. For further information, please see our editorial policies site. All relevant questions on the form must be answered.

Competing interests

Policy information about competing interests

In the interest of transparency and to help readers form their own judgements of potential bias, Nature Portfolio journals require authors to declare any competing financial and/or non-financial interest in relation to the work described in the submitted manuscript.

Competing interests declaration

- [ ] We declare that none of the authors have competing financial or non-financial interests as defined by Nature Portfolio.
- [x] We declare that one or more of the authors have a competing interest as defined by Nature Portfolio.

J.H.C. and Y.L.W. have financial interests in NanoAffix Science LLC. The company did not fund this work. All other authors declare no competing financial interests.

Authorship

Policy information about authorship

Prior to submission all listed authors must agree to all manuscript contents, the author list and its order and the author contribution statements. Any changes to the author list after submission must be approved by all authors.

- [x] We have read the Nature Portfolio Authorship Policy and confirm that this manuscript complies.

Policy information about Authorship: inclusion & ethics in global research

All authors are encouraged to provide an "Inclusion & Ethics" statement where relevant.

- [ ] We have provided an "Inclusion & Ethics" statement.

Data availability

Policy information about availability of data

Data availability statement

All manuscripts must include a data availability statement. This statement should provide the following information, where applicable:

- Accession codes, unique identifiers, or web links for publicly available datasets
- A description of any restrictions on data availability
- For clinical datasets or third party data, please ensure that the statement adheres to our policy

- [x] We have provided a full data availability statement in the manuscript.

Mandated accession codes (where applicable)

Confirm that all relevant data are deposited into a public repository and that accession codes are provided.

- [ ] All relevant accession codes are provided
- [ ] Accession codes will be available before publication
- [x] No data with mandated deposition

Code availability

Policy information about availability of computer code

Code availability statement

For all studies using custom code or mathematical algorithm that is deemed central to the conclusions, the manuscript must include a statement under the heading "Code availability" describing how readers can access the code, including any access restrictions. Code availability statements should be provided as a separate section after the data availability statement but before the References.

- [ ] We have provided a full code availability statement in the manuscript

Data presentation

For all data presented in a plot, chart or other visual representation confirm that:

n/a	Confirmed	
☐	☒	Individual data points are shown when possible, and always for $n \leq 10$
☐	☒	The format shows data distribution clearly (e.g. dot plots, box-and-whisker plots)
☒	☐	Box-plot elements are defined (e.g. center line, median; box limits, upper and lower quartiles; whiskers, 1.5x interquartile range; points, outliers)
☐	☒	Clearly defined error bars are present and what they represent (SD, SE, CI) is noted

Image integrity

Policy information about image integrity

☒ We have read Nature Portfolio's image integrity policy and all images comply.

Unprocessed data must be provided upon request. Please double-check figure assembly to ensure that all panels are accurate (e.g. all labels are correct, no inadvertent duplications have occurred during preparation, etc.).

Where blots and gels are presented, please take particular care to ensure that lanes have not been spliced together, that loading controls are run on the same blot, and that unprocessed scans match the corresponding figures.

Additional policy considerations

Some types of research require additional policy disclosures. Please indicate whether each of these apply to your study. If you are not certain, please read the appropriate section before selecting a response.

Does not apply	Involved in the study	
☒	☐	Macromolecular structural data
☒	☐	Unique biological materials
☒	☐	Research animals and/or animal-derived materials that require ethical approval
☒	☐	Human embryos, gametes and/or stem cells
☒	☐	Human research participants
☒	☐	Clinical data
☒	☐	Archaeological, geological, and palaeontological materials

I certify that all the above information is complete and correct.

Typed signature Junhong Chen Date Jun 12, 2023

This checklist template is licensed under a Creative Commons Attribution 4.0 International License, which permits use, sharing, adaptation, distribution and reproduction in any medium or format, as long as you give appropriate credit to the original author(s) and the source, provide a link to the Creative Commons license, and indicate if changes were made. The images or other third party material in this article are included in the article's Creative Commons license, unless indicated otherwise in a credit line to the material. If material is not included in the article's Creative Commons license and your intended use is not permitted by statutory regulation or exceeds the permitted use, you will need to obtain permission directly from the copyright holder. To view a copy of this license, visit http://creativecommons.org/licenses/by/4.0/
